# 血浆EB病毒DNA载量监测对单倍体造血干细胞移植后淋巴细胞增殖性疾病的预测价值

**DOI:** 10.3760/cma.j.issn.0253-2727.2023.04.004

**Published:** 2023-04

**Authors:** 静 陈, 于谦 孙, 兰平 许, 晓辉 张, 开彦 刘, 晓冬 莫, 翼飞 程, 晓军 黄, 昱 王

**Affiliations:** 北京大学人民医院，北京大学血液病研究所，国家血液系统疾病临床医学研究中心，造血干细胞移植北京市重点实验室，北京 100044 Peking University People's Hospital, Peking University Institute of Hematology, National Clinical Research Center for Hematologic Disease, Beijing Key Laboratory of Hematopoietic Stem Cell Transplantation, Beijing 100044, China

**Keywords:** 单倍体造血干细胞移植, 移植后淋巴增殖性疾病, EB病毒, 预测, Haploidentical hematopoietic stem cell transplantation, Post-transplant lymphoproliferative disease, Epstein-Barr virus, Predict

## Abstract

**目的:**

寻找可以协助诊断单倍体造血干细胞移植（haplo-HSCT）后淋巴细胞增殖性疾病（PTLD）的EB病毒DNA（EBV-DNA）载量及其持续时间的最佳临界值。

**方法:**

回顾性分析北京大学血液病研究所2016年1月至12月期间haplo-HSCT后合并EBV感染患者的相关数据，通过构建ROC曲线计算约登指数寻找对PTLD有诊断意义的EBV-DNA载量及其持续时间的临界值。

**结果:**

共纳入94例患者，其中20例（21.3％）发生PTLD，诊断PTLD时中位EBV-DNA载量为70 400（1 710～1370 000）拷贝/ml，EBV血症中位持续时间为23.5（4～490）d。二元logistic回归分析显示，PTLD组与非PTLD组两组间EBV-DNA最高载量及EBV血症持续时间差异均有统计学意义（*P*＝0.018，*P*＝0.001）。构建ROC曲线计算约登指数，EBV-DNA载量≥41 850拷贝/ml对PTLD有诊断意义［曲线下面积（AUC）＝0.847］，敏感度、特异度分别为0.611、0.932；EBV血症持续时间≥20.5 d对PTLD有诊断意义（AUC＝0.833），敏感度、特异度分别为0.778、0.795。

**结论:**

动态监测haplo-HSCT后PTLD高危患者的EBV载量及关注其持续时间有重要临床意义，有助于预测PTLD的发生并早期采取干预措施。

## Abstract (en)

**Objective:**

To determine the optimal cutoff value of Epstein-Barr virus（EBV）DNA load that can assist in the diagnosis of post-transplant lymphoproliferative disease（PTLD）after haploidentical hematopoietic stem cell transplantation（haplo-HSCT）.

**Methods:**

The data of patients with EBV infection after haplo-HSCT from January to December 2016 were retrospectively analyzed. Through constructing the receiver operating characteristic（ROC）curve and calculating the Youden index to determine the cutoff value of EBV-DNA load and its duration of diagnostic significance for PTLD.

**Results:**

A total of 94 patients were included, of whom 20（21.3％）developed PTLD, with a median onset time of 56（40–309）d after transplantation. The median EBV value at the time of diagnosis of PTLD was 70,400（1,710–1,370,000）copies/ml, and the median duration of EBV viremia was 23.5（4–490）d. Binary logistic regression was used to analyze the peak EBV-DNA load（the EBV-DNA load at the time of diagnosis in the PTLD group）and duration of EBV viremia between the PTLD and non-PTLD groups. The results showed that the difference between the two groups was statistically significant（*P*＝0.018 and *P*＝0.001）. The ROC curve was constructed to calculate the Youden index, and it was concluded that the EBV-DNA load ≥ 41 850 copies/ml after allogeneic hematopoietic stem cell transplantation had diagnostic significance for PTLD（AUC＝0.847）, and the sensitivity and specificity were 0.611 and 0.932, respectively. The duration of EBV viremia of ≥20.5 d had diagnostic significance for PTLD（AUC＝0.833）, with a sensitivity and specificity of 0.778 and 0.795, respectively.

**Conclusion:**

Dynamic monitoring of EBV load in high-risk patients with PTLD after haplo-HSCT and attention to its duration have important clinical significance, which can help clinically predict the occurrence of PTLD in advance and take early intervention measures.

异基因造血干细胞移植（allo-HSCT）是恶性血液系统疾病的重要治疗方法。移植后淋巴细胞增殖性疾病（PTLD）作为allo-HSCT后严重并发症，近些年来也受到了越来越多的关注，由于PTLD是一组异质性疾病、临床表现复杂多样、确诊需要有创性检查手段等导致该疾病诊断困难。若其早期未发现，则会迅速发展，死亡率极高[1]–[3]。allo-HSCT后PTLD几乎均由EB病毒（EBV）再激活所致，EBV阴性PTLD罕见[4]–[5]。许多研究证实PTLD的发生与外周血中EBV-DNA拷贝数的快速增多有关联，EBV-DNA载量越高发生PTLD的风险越大[6]–[8]。如果找到能协助诊断PTLD的EBV-DNA载量或其持续时间的最佳临界值，可以给临床诊断PTLD并及早开始治疗提供便利，进而改善患者预后，特别是对于无法获得活检组织标本的患者[9]。目前相关研究较少，特别是针对单倍体造血干细胞移植（haplo-HSCT）患者。

本研究即通过回顾性分析94例haplo-HSCT后合并EBV血症患者的临床数据探讨EBV-DNA载量及其持续时间能否协助诊断PTLD。

## 病例与方法

一、病例资料

采用回顾性分析方法，选取自2016年1月至2016年12月于北京大学血液病研究所进行异基因造血干细胞移植并合并EBV血症患者105例。符合纳入及排除标准的haplo-HSCT患者94例，其中排除了1例数据严重缺失患者及1例临床诊断待定患者。

纳入标准：所有急性淋巴细胞白血病（ALL）、急性髓系白血病（AML）、重型再生障碍性贫血（SAA）、骨髓增生异常综合征（MDS）等进行haplo-HSCT后合并EBV血症的患者。

PTLD的诊断参照欧洲白血病抗感染学会（ECIL-6）标准[4]，包括临床诊断和确定诊断。临床诊断标准：①存在发热、淋巴结肿大、肝脾肿大或其他终末器官病变等临床表现。②外周血中EBV-DNA拷贝数定量阳性。③缺乏其他合理的病因解释；确定诊断标准：除包括临床诊断标准外，还包括组织活检标本发现原有细胞结构被增生的淋巴组织破坏并检测到EBV核酸或蛋白或组织病理学具有PTLD特征（淋巴发育过程受阻、存在单克隆淋巴细胞等）。排除标准：亲缘全相合移植患者、非血缘移植患者、重要数据缺失及临床诊断待定患者（如无法确定是否合并PTLD）。

本组病例对EBV血症的治疗顺序按照密切观察、免疫抑制剂减量、利妥昔单抗治疗、细胞疗法而逐级递增。

二、分组与观察指标

按照患者是否发生PTLD将患者分为PTLD组和非PTLD组，分别包含20例、74例患者。

对所有入选患者的住院病历进行分析、归纳并记录以下资料：①患者一般资料：姓名、性别、年龄、原发病。②实验室及影像学检查结果：移植后EBV-DNA载量（包括EBV-DNA开始阳性时间、EBV-DNA转阴时间、EBV血症持续时间、EBV-DNA最高载量、诊断PTLD时EBV-DNA载量等）。该研究中的EBV-DNA载量是运用ABI 7500荧光定量PCR仪采用实时荧光定量（realtime PCR）的方法检测血浆中的EBV-DNA拷贝数，≥500拷贝/ml为阳性。一般在allo-HSCT后1个月内开始检测，每周至少1次，直到移植后4个月。若发现EBV-DNA阳性，则每周检测2次，并延长监测时间。③记录患者治疗情况（移植类型、药物）、是否干预EBV血症、有无发生PTLD、PTLD发病时间、PTLD类型、诊断依据、治疗、转归等。

三、统计学处理

应用SPSS 23.0软件进行数据分析，两组间临床特征采用非参数检验或Fisher精确概率法进行比较，采用二元logistic回归方法分析影响PTLD发生的独立危险因素。*P*值<0.05认为差异有统计学意义。构建受试者工作特征曲线（ROC曲线）确定EBV-DNA载量和EBV血症持续时间诊断PTLD的最佳临界值、敏感度、特异度及ROC曲线下面积。

## 结果

一、基本临床资料

共纳入94例haplo-HSCT后合并EBV血症患者，其中男61例，女33例，中位年龄为26.5（5～49）岁。基础疾病包括ALL 43例、AML 32例、MDS 7例，SAA 5例，其他疾病7例。应用非参数检验对两组间的基本临床特征进行比较，结果显示两组间年龄、性别、原发病构成差异均无统计学意义（表1）。

**表1:** 94例haplo-HSCT后合并EB病毒血症血液病患者中发生、未发生PTLD两组患者基本临床资料特征比较分析

基本特征	PTLD组（20例）	非PTLD组（74例）	*P*值
年龄［岁，*M*（*P*25，*P*75）］	26.5（22.2，28.7）	27.5（19.5，35.0）	0.621
性别［例（%）］			0.113
男	16（80.0）	45（60.8）	
女	4（20.0）	29（39.2）	
原发病（例）			0.266
ALL	10	33	
AML	6	26	
MDS	2	5	
AA	0	5	
其他	2	5	

**注** haplo-HSCT：单倍体造血干细胞移植；PTLD：移植后淋巴细胞增殖性疾病；ALL：急性淋巴细胞白血病；AML：急性髓系白血病；MDS：骨髓增生异常综合征；AA：再生障碍性贫血

二、诊疗情况

预处理方案均含抗胸腺细胞免疫球蛋白（ATG）。94例患者均合并EBV血症，诊断后16例患者未干预，34例患者给予免疫抑制剂减量，38例患者给予利妥昔单抗治疗，4例患者给予EBV特异性细胞毒性T淋巴细胞（EBV-CTL）治疗。

三、PTLD患病情况

20例（21.3％）发生了PTLD，不同EBV-DNA载量组PTLD发生率详见表2。其中4例为确诊诊断病例，16例为临床诊断病例。PTLD中位发病时间为移植后56（40～309）d。

**表2:** 不同EBV-DNA载量组移植后淋巴增殖性疾病（PTLD）发生率的比较

EBV-DNA载量（拷贝/ml）	例数	发生PTLD［例（%）］
<4000	43	2（4.7）
4000~40 000	34	6（17.6）
>40 000~400 000	14	9（64.3）
>400 000	3	3（100）

总计	94	20（21.3）

**注** EBV：EB病毒

四、EBV-DNA载量相关

PTLD组20例患者诊断PTLD时中位EBV-DNA载量为70 400（1 710～1370 000）拷贝/ml，EBV血症中位持续时间为23.5（4～490）d。非PTLD组74例患者中位EBV-DNA最高载量为3 435（515～334 000）拷贝/ml，EBV血症中位持续时间为10（2～46）d。应用二元logistic回归分析显示两组间EBV-DNA最高载量（若诊断则用诊断时EBV-DNA载量）及EBV血症持续时间差异均具有统计学意义（*P*＝0.018，*P*＝0.001）。构建ROC曲线计算约登指数，当EBV-DNA载量为41 850拷贝/ml时，ROC曲线下面积最大（0.847），敏感度、特异度分别为0.611、0.932；当EBV持续阳性20.5 d时，ROC曲线下面积最大（0.833），敏感度、特异度分别为0.778、0.795。联合EBV-DNA拷贝数及持续时间生成新变量绘制四格表，若EBV-DNA载量大于等于41 850拷贝/ml或EBV血症持续时间≥20.5 d诊断PTLD的敏感度、特异度分别为0.850、0.730（*P*<0.001）；若两者同时符合，敏感度、特异度分别为0.400、1.000（*P*＝0.999，*P*>0.05）。

五、利妥昔单抗治疗患者的结局

本研究中38例EBV血症患者接受了利妥昔单抗治疗，34例患者EBV-DNA转阴并持续，2例患者EBV-DNA转阴后复阳，2例患者EBV-DNA持续阳性，有效率为89.5％（34/38）。13例发生PTLD的患者中8例经过继续接受利妥昔单抗等治疗后病情好转，5例（38.5％）病情恶化死亡。

六、PTLD患者的预后

本研究中20例PTLD患者诊断后均接受了以利妥昔单抗为基础的治疗，14例患者EBV-DNA转为阴性，2例患者EBV-DNA复阳，4例患者EBV-DNA持续阳性，有效率为70％（14/20）。10例PTLD患者治疗后病情好转，10例（50％）患者死亡（2例死于原发病复发，8例死于严重感染）。

## 讨论

由于haplo-HSCT需要使用ATG预防GVHD且免疫抑制剂使用时间更长，因此发生PTLD的风险高于其他移植模式[5]。本研究共纳入94例EBV血症患者，其中20例发生PTLD，发生率为21.3％，国内相关报道中haplo-HSCT合并EBV感染后的PTLD发生率为34.6％[10]。本研究中20例PTLD患者中4例为确诊，16例为临床诊断，与Xu等[11]报道的构成比相似（确诊26.7％、临床诊断73.3％）。确诊病例占比较低与PTLD需要有创性检查相关，但大部分患者获得活检组织标本困难，而且等待组织学证据有可能错过PTLD的最佳治疗时机，这也是我们设计本研究的原因之一。

EBV再激活和免疫监视受损是PTLD发病机制中的关键因素[12]。移植患者体内潜伏的EBV可源自患者或供者，在移植后免疫抑制状态下重新激活。研究证实EBV再激活为PTLD发生的独立危险因素，随着EBV-DNA载量的增加，PTLD发生率逐步增加[8]。研究提示高循环水平的EBV负荷可被用作PTLD的标志物[9]。但目前仍尚无诊断PTLD的外周血EBV DNA载量临界值水平的共识[2],[9]。动态监测EBV-DNA载量持续增加可预测PTLD的发生，但具体时间亦少有文章阐述。Kinch等[3]研究发现将血浆EBV-DNA水平1000 gEq/ml作为诊断PTLD的阈值敏感度只有28％～39％。牛燕燕等[8]研究提示当全血EBV载量为1.19×10^6^拷贝/ml时，ROC曲线下面积最大（0.774），敏感度为0.800，特异度为0.768。

临床中检测EBV-DNA载量可选择的标本类型有全血、血浆及外周血单个核细胞。研究发现，血浆标本在评估EBV相关性疾病及追踪治疗反应方面更准确[13]。本研究证实血浆EBV-DNA载量可以作为PTLD的诊断标志物，最佳临界值为41 850拷贝/ml，敏感度为0.611，特异度为0.932，特异度优于牛燕燕等[8]研究中的全血标本。同时证实EBV血症持续时间对PTLD也有诊断意义，最佳临界值为20.5 d，敏感度为0.778，特异度为0.795。两者并联可以提高敏感度，且结果有统计学意义，两者串联可以提高特异度，但结果尚未达到统计学意义。

对于启动抢先治疗的EBV-DNA拷贝数定量目前无公认的推荐阈值[4],[14]–[16]。有研究推荐将1×10^3^拷贝/10^6^外周血单个核细胞[16]、血浆EBV-DNA 1×10^3^拷贝/ml[17]、全血EBV-DNA 1×10^4^ 拷贝/ml[18]作为利妥昔单抗抢先治疗的阈值。本研究中EBV-DNA载量4 000～40 000拷贝/ml时PTLD发生率（17.6％）与整体PTLD发生率（21.3％）大致相当，当高于此水平时PTLD发生率明显增高，可考虑将血浆EBV-DNA载量4 000拷贝/ml作为利妥昔单抗抢先治疗的阈值。

不同研究中心EBV-DNA载量测定的标本类型、仪器、试剂盒等不尽相同，因此各中心检测结果无法直接比较，应尽快制定标准检测方法至关重要。本研究中的EBV-DNA载量是由ABI 7500荧光定量PCR仪通过实时荧光定量的方法检测血浆标本得出，若检测仪器及标本一致可供参考。

综上所述，PTLD对患者的生存质量和预后均有严重不良影响，监测EBV-DNA载量对PTLD的发生有预测价值，有助于早期干预进而改善预后。
